# MicroRNAs in Uterine Leiomyosarcoma: From Molecular Mechanisms to Clinical Applications

**DOI:** 10.3390/ijms262210952

**Published:** 2025-11-12

**Authors:** Areti Kourti, Ioannis Kalogiannidis, Kali Makedou, Elisavet Georgiou

**Affiliations:** 1 Laboratory of Biological Chemistry, School of Medicine, Faculty of Health Sciences, Aristotle University of Thessaloniki, GR-54124 Thessaloniki, Greece; aretikourti@auth.gr (A.K.); elgeorgiou@auth.gr (E.G.); 2 3rd Department of Obstetrics and Gynecology, School of Medicine, Faculty of Health Sciences, Aristotle University of Thessaloniki, GR-54124 Thessaloniki, Greece; ikalogia@auth.gr; 3 Laboratory of Biochemistry, School of Medicine, Faculty of Health Sciences, Aristotle University of Thessaloniki, GR-54124 Thessaloniki, Greece

**Keywords:** microRNAs, uterine leiomyosarcoma, biomarkers, tumor-suppressor genes, oncogenic pathways, prognosis, targeted therapy, precision oncology

## Abstract

Uterine leiomyosarcoma (uLMS) is a rare, highly aggressive malignancy of uterine smooth muscle, associated with early metastasis, frequent recurrence, and poor prognosis. Accurate preoperative diagnosis remains difficult given that clinical and radiologic features often overlap with benign leiomyomas, and no reliable biomarkers are currently available. This review summarizes recent evidence on the role of microRNAs (miRNAs) in the biology and clinical management of uLMS. Literature from molecular and translational studies was examined to identify dysregulated miRNAs, their target pathways, and potential diagnostic and therapeutic applications. uLMS displays a characteristic miRNA profile, including downregulation of tumor-suppressive miRNAs such as the miR-29 and miR-200 families and upregulation of oncogenic miRNAs including miR-21 and the miR-183~96~182 cluster, leading to activation of PI3K/AKT/mTOR signaling and epithelial–mesenchymal transition (EMT). Circulating and tissue miRNAs show promise as minimally invasive biomarkers for differentiating uLMS from leiomyomas, predicting prognosis, and guiding therapy. Emerging therapeutic approaches aim to restore the tumor-suppressive miRNAs or inhibit oncogenic ones using mimics or antagomiRs. Overall miRNAs represent critical regulators of uLMS pathogenesis and hold significant potential for precision diagnosis, prognostication, and targeted therapy, though larger validation studies and improved delivery systems are required before clinical translation.

## 1. Introduction

Uterine Leiomyosarcoma (uLMS) is the most common malignant tumor targeting uterine smooth muscle and is characterized by highly aggressive biological behavior, frequent recurrences and poor prognosis compared to other gynecologic malignancies [1]. Despite advances in surgical treatment and systemic therapy, five-year survival rates remain dismal, especially in advanced stages [2]. Diagnosis of uLMS is often challenging, since it can mimic the clinical and radiologic manifestations of benign leiomyomas, and a substantial proportion of cases are detected incidentally following hysterectomy or myomectomy [3]. At a molecular level, common alterations in *TP53*, *RB1*, *ATRX*, and *MED12*, along with profound genomic instability, have been reported [4], thus highlighting the need and potential opportunities for targeted treatment. Nevertheless, the absence of reliable biomarkers continues to limit early detection, risk stratification and therapeutic guidance. In light of this, microRNAs (miRNAs) have emerged as key regulators of gene expression, with established roles in tumorigenesis and advancing prospect as minimally invasive tools for diagnosis, prognosis, and even targeted treatment in cancer [5]. Therefore, this review summarizes the key concepts surrounding the role of miRNAs as possible biomarkers in addressing the challenges faced in the detection, staging and management of uLMS.

## 2. Uterine Leiomyosarcoma (uLMS)

### 2.1. Overview and Epidemiology

Uterine Leiomyosarcoma is a rare, aggressive malignant tumor that arises from smooth muscle cells of the myometrium and accounts for approximately 60–70% of all uterine sarcomas [6]. From a histological point of view, it is characterized by a combination of diffuse cytologic atypia, brisk mitotic activity, and tumor cell necrosis [3]. Unlike benign leiomyomas, uLMS demonstrates rapid cancer cell proliferation, early hematogenous dissemination and a high tendency for recurrence even after complete surgical treatment [7].

The incidence rate of uLMS ranges from 0.36 to 0.64 per 100,000 women per year, marking it epidemiologically exceedingly rare [8]. Population-based studies show that the median age of women diagnosed with uLMS is between 50 and 55 years, with the disease being most common in peri- and postmenopausal women [8,9]. The prevalence of unsuspected uLMS in women undergoing surgery for presumed leiomyomas is approximately 0.2–0.4% and considered low but clinically concerning, given the significant risk of accidental tumor dissemination [10]. Despite aggressive management and overall disease approach, the five-year survival remains poor, ranging from 18.8% to 65% with a recurrence rate ranging from 53% to 71% [11].

### 2.2. Risk Factors

Only a few risk factors have been associated with increased incidence of uLMS. The identified and documented risk factors include hereditary retinoblastoma, Li-Fraumeni syndrome (inherited TP53 mutations), prior pelvic exposure to radiation, and the therapeutic administration of tamoxifen, while other potential contributors remain poorly defined [10,12]. The scarcity of established risk factors underlines the difficulty of early detection of uLMS and highlights the need for novel molecular biomarkers.

### 2.3. Clinical Presentation and Symptoms

The clinical features of uLMS are often non-specific and overlap significantly with those of benign leiomyomas, thus contributing to frequent delays in diagnosis. Abnormal uterine bleeding is reported in 50–70% of newly diagnosed uLMS, while pelvic or abdominal pain and a rapidly enlarging uterine mass are included among the most common symptoms [9,13]. Constitutional symptoms such as fatigue, weight loss, or anemia may also appear in advanced disease [14]. Imaging techniques, particularly ultrasound and magnetic resonance imaging (MRI) cannot always distinguish uLMS from benign leiomyomas, given the frequency that this malignancy mimics the radiologic image of leiomyomas. Nevertheless, features such as heterogenous echotexture, irregular margins, central necrosis, and increased vascularity or neoangiogenesis may raise suspicion for uLMS [15]. Ultimately, around 0.2–0.4% of patients undergoing surgical treatment for alleged benign leiomyomas are incidentally diagnosed with uLMS, thus highlighting the diagnostic challenge [10]. The absence of pathognomonic clinical or radiologic signs generally makes the histopathologic examination of the surgical specimen a required step towards a definitive diagnosis [14].

### 2.4. Histopathology and Differential Diagnosis

The classic triad of diffuse cytologic atypia, high mitotic index, and tumor cell necrosis defines histologically uLMS [3,16]. The 2020 World Health Organization classification of female genital tumors emphasizes the essential value of these criteria in distinguishing uLMS from other smooth muscle tumors of the uterus [17]. Typically, tumors present with pleomorphic spindle-shaped cells containing hyperchromatic nuclei, a mitotic activity of more than 10 mitoses per 10 high-power fields (HPF), and coagulative tumor cell necrosis characterized by abrupt transition between viable and necrotic tumor tissue [18]. From an immunohistochemical lens, uLMS usually expresses smooth muscle markers such as desmin, smooth muscle actin (SMA), and h-caldesmon, while often a loss of expression of estrogen and progesterone receptors (ER and PR) is present [19]. At a molecular level, recurrent alterations including, but not limited to, TP53, RB1, PTEN, ATRX, and MED12 are commonly observed, mirroring the high genomic instability of this tumor type [4].

The challenge in the differential diagnosis of uLMS stems from the initial presentation of patients with a uterine mass suspicious for a benign leiomyoma. Leiomyoma, the most common benign tumor of the uterine smooth muscle (Table 1) tissue, can share overlapping clinical and radiologic features with uLMS, especially when presented with atypical or degenerative changes [13]. Histologically, leiomyomas typically lack significant cytologic atypia, have a low mitotic activity of less than 5 mitoses per 10 HPF, and exhibit only ischemic-type tumor cell necrosis [20]. An interesting variant of leiomyomas is that of mitotically active leiomyomas, which show benign clinical behavior and a low risk of metastasis [21]. An additional significant consideration in the differential diagnosis is Smooth Muscle Tumors of Uncertain Malignant Potential (STUMP), which demonstrate features that cannot be unequivocally classified as benign or malignant. STUMPs may present with localized cell atypia or increased mitotic activity but fail to meet all three histological diagnostic criteria for uLMS [22]. Given this wide yet uncertain diagnostic spectrum, accurate classification requires thorough assessment of all histopathologic parameters, supplemented by immunohistochemistry and, in some cases, molecular analysis, hence the need for novel biomarkers.

### 2.5. Molecular and Genetic Landscape

In contrast to benign leiomyomas, uLMS is characterized by a high degree of genomic instability and a distinctive mutational landscape. The most frequently altered gene is *TP53*, which is found mutated or deleted in up to 60–80% of cases, leading to the loss of cell cycle regulation. Inactivation of RB1 gene, often through deletions, occurs in approximately 50% of tumors and contributes to uncontrolled cell proliferation [4]. Additionally, mutations or loss of protein expression in ATRX gene are reported in 25–35% of cases and are associated with alternative lengthening of telomeres (ALT), a mechanism of genomic maintenance which is linked to poor prognosis [23,24]. Other common alterations, though less frequent than *TP53* and *RB1*, include mutations in *PTEN*, *MED12*, *CDKN2A*, and PI3K/AKT/mTOR pathway genes [4,14]. Overall, these molecular findings accentuate the central role of tumor suppressor gene dysfunction in the pathogenesis of uLMS.

Cytogenetically, uLMS reveals complex, highly aneuploid karyotypes with frequent chromosomal additions and deletions. Common abnormalities include deletions at chromosome 10q (*PTEN* locus) and 13q14 (*RB1* locus), and additions in 1q and 17p, regions containing genes that are involved in cell cycle regulation, progression and apoptosis [1,25]. Transcriptomics and other pathway analyses have highlighted the dysregulation of the p53/RB pathway, PI3K/AKT/mTOR signaling, and genes related to DNA damage response and chromatin remodeling [14]. Epigenetic deregulation, including altered DNA methylation patterns and miRNA expression, further contributes to the aggressive phenotype of uLMS [1]. The conjunction of genetic and chromosomal abnormalities in cell cycle regulation and DNA repair pathways expounds both the aggressive behavior and therapeutic resistance of uLMS, while also indicating potential targets for novel therapies.

### 2.6. Pathophysiology

A convergence of genomic instability, disrupted signaling networks, and microenvironmental interactions steers the pathophysiology of uLMS. A key feature is loss of cell cycle regulation and DNA repair capacity, which stimulates uncontrolled proliferation and tumor heterogeneity [25]. Indefinite replicative immortality of cells is usually supported by ALT following the loss of ATRX gene [24]. Beyond innate tumor genetics, the tumor microenvironment (TME) plays a key role: uLMS often presents with a low lymphocytic infiltration phenotype, whereas the activation of PI3K/mTOR pathway leads to immune evasion. A recent preclinical study indicates that PI3K/mTOR pathway inhibition remodels the TME, enhances antigen presentation and sensitizes tumors to PD-1 blockade, thus highlighting the interchange between oncogenic signaling and immune suppression [26]. In addition, epigenetic deregulation—including DNA methylation signatures and dysregulated miRNAs—further contributes to the reprogramming of transcriptional networks and distinguishes uLMS from leiomyosarcomas at other anatomical sites [27]. Comparative studies of metastatic disease show that specific genetic variants, such as *TP53* alteration, are enhanced in aggressive short-term survivors, underscoring the relationship between molecular drivers and clinical behavior [28]. Altogether, these findings showcase that uLMS progression arises not only from tumor suppressor loss, but also from dynamic interactions between tumor genetics, epigenetics and TME.

### 2.7. Diagnosis and Staging

The majority of uLMS cases are diagnosed postoperatively, when a surgically resected specimen reveals through histopathologic examination the diagnostic triad of cytologic atypia, mitotic index, and tumor cell necrosis [18]. Preoperative radiologic imaging with ultrasound and MRI can indicate suspicious features, such as irregular margins, heterogeneity in signal intensity, areas of hemorrhage or necrosis, and increased vascularity, yet they cannot provide a definitive answer for a secure diagnosis [29,30]. Serum markers such as LDH isoenzymes and CA-125 have been studied, but none are validated for routine clinical use [31]. Therefore, accurate diagnosis relies primarily on histopathologic and immunohistochemical evaluation of the uterine mass following surgery.

Staging of uLMS is implemented according to the 2009 FIGO staging system for uterine sarcomas, which provides a detailed categorization for the disease based on tumor size, anatomical site, and metastasis [31,32]. Stage I uLMS is confined to the uterus and subdivided into IA (<5 cm) an IB (>5 cm). Stage II represents extrauterine pelvic extension, whereas stage III indicates abdominal involvement, and stage IV includes invasion of the bladder, rectum, or distant metastases [33]. Surgical exploration is required to perform accurate staging, given that imaging may underestimate extrauterine dissemination. This is significant, as uLMS is usually diagnosed at an advanced stage due to its aggressive behavior, with up to 50% of patients presenting with extrauterine disease extension at the time of diagnosis [14,33]. Staging not only guides the prognosis, but also plays a vital role in treatment strategies, therapeutic targets, and surveillance planning.

### 2.8. Prognostic and Predictive Factors

In comparison to other uterine malignancies, uLMS carries a dismal prognosis, with five-year survival rates ranging from 25% to 60%, strongly influenced by the tumor stage at the time of diagnosis (Table 2) [14]. Despite complete surgical resection, recurrence rates reach 50–70%, most commonly with metastases to lung, liver, or peritoneum [34]. The most invariably validated clinicopathologic prognostic markers include tumor stage and size, mitotic index, and the presence of coagulative tumor cell necrosis, while molecular features such as gene alterations have been linked with worse outcomes [28,35]. Tumors larger than 10 cm and those with high mitotic activity pose a significantly higher risk of recurrence and disease-specific mortality [36]. Survival is also affected by certain patient characteristics such as older age and poor performance status [37]. At a molecular level, alterations in *TP53*, *RB1* and *ATRX* have been linked to more aggressive disease biology and worse progression-free survival [28]. Epigenetic deregulation, including distinct DNA methylation patterns, is emerging as an additional layer of prognostic information, further differentiating uLMS from other leiomyosarcomas and correlating with disease aggressiveness and progression [27].

Predictive biomarkers that can guide therapy selection in uLMS still remain under investigation. Hormone receptor status (ER and PR) has been studied as a predictor of the response to endocrine therapy, without producing consistent results [35]. Recently, immune microenvironment features have shown promise, as tumors with low T-cell infiltration and activation of the PI3K/mTOR pathway appear to be resistant to immune checkpoint inhibition. In parallel, preclinical evidence suggests that blocking the PI3K/mTOR pathway can remodel the TME and sensitize tumors to PD-1 inhibitors [26]. Expression profiles of miRNAs are equally under study as predictors of both treatment response and resistance, especially in relation to targeted therapies [27]. While no predictive marker is yet validated for routine clinical use, the incorporation of molecular and immune biomarkers in the traditional clinicopathologic factors demonstrates a key future direction for improving therapeutic stratification in uLMS.

### 2.9. Treatment

Surgery is the cornerstone of treatment for localized uLMS. Conventional management is total hysterectomy with en bloc resection of the mass, avoiding tumor fragmentation. A current tailored approach is bilateral oophorectomy, while lymphadenectomy is not routinely indicated given the low incidence of nodal disease [38,39]. Uncontained power morcellation of presumed fibroids enhances the risk of intraperitoneal dissemination of undetected uLMS, therefore its use is generally discouraged [40].

The benefit of adjuvant therapy remains uncertain. Radiotherapy may reduce local recurrence but does not improve overall survival, so its use is selective [41]. Similarly, the survival benefit of adjuvant chemotherapy is inconsistent, and its use is generally reserved for high-risk patients or clinical trials [38]. For advanced or recurrent disease, systemic chemotherapy remains the standard of care. Doxorubicin is a first-line option, with or without ifosfamide, while gemcitabine plus docetaxel is widely used in leiomyosarcoma, and other common agents include trabectedin, dacarbazine, and pazopanib, with modest activity [42]. Long-lasting responses to treatment are rare, hence the need for better systemic therapeutic strategies.

Targeted and immune therapies are currently under investigation. Frequent activation of the PI3K/mTOR pathway contributes to tumor growth and immune invasion; inhibition of this pathway has shown to transform the TME and sensitize uLMS to PD-1 blockade in preclinical models [26]. Clinical trials are exploring possible combinations of immunotherapy with chemotherapy or targeted agents, as well as PARP inhibitors and other approaches in biomarker-selected patients [43].

### 2.10. Current Challenges in Management

The management of uLMS is hindered by several persistent challenges. At the diagnostic level, accurate preoperative identification remains elusive, as current imaging techniques and biomarkers cannot safely distinguish uLMS from benign leiomyomas, often leading to incidental diagnosis after surgery [30]. Regarding adjuvant therapy, even though chemotherapy and radiotherapy may provide local control or delay recurrence, they do not hold a consistent survival advantage since many patients are vulnerable to systemic relapse [38,41]. In advanced and recurrent disease, cytotoxic chemotherapy offers only modest response rates and short progression-free intervals, with no current regimen providing an ultimate survival benefit [42]. Median OS for patients with metastatic disease is typically 12–24 months, and long-term survival in this group remains scarce [33]. These inauspicious statistics highlight the aggressive biology of uLMS and the need for improved treatment strategies. From a research standpoint, progress is hindered by the rarity of the disease, which complicates patient recruitment for large randomized controlled trials and slows the validation of promising molecular or immune-based strategies [1]. As far as surgical practice is concerned, the constant risk of tumor dissemination associated with power morcellation, emphasizes the need for both technical caution and regulatory surveillance [40]. Conjointly, these challenges shed light on the urgency of developing effective biomarkers, novel systemic treatments and international collaborative trials to improve outcomes in this rare and aggressive malignancy.

## 3. microRNAs (miRNAs)

### 3.1. Definition and Biogenesis

MicroRNAs (miRNAs) are a type of small, non-coding RNAs comprising approximately 18–25 nucleotides that regulate gene expression at the post-transcriptional level. They carry out their function by binding to complementary sequences in target messenger RNAs (mRNAs), leading to translational repression or degradation, thus controlling diverse biological processes including proliferation, differentiation, apoptosis and tumorigenesis [44,45]. The biogenesis of miRNAs is a multistep process. It begins in the nucleus with the transcription of primary miRNAs (pri-miRNAs) by RNA polymerase II, which are further processed by the Drosha-DGCR8 microprocessor complex into precursor miRNAs (pre-miRNAs). These 70 nucleotide-long hairpin structures are exported to the cytoplasm with the help of Exportin-5, where they undergo further cleavage by Dicer, thus producing mature double-stranded miRNA duplexes. One strand (guide) is incorporated into the RNA-induced Silencing Complex (RISC), where it directs target recognition, while the other strand (passenger) is typically degraded [46,47]. This finely tuned process is critical for maintaining homeostasis, and its dysregulation contributes to oncogenesis in numerous tumor types, including leiomyosarcoma.

### 3.2. Functional Roles of miRNAs

MicroRNAs function as key regulators of gene expression, influencing a wide range of biological processes including cell proliferation, differentiation, apoptosis, angiogenesis and immune response. Their functional roles in cancer can be broadly divided into two categories: oncogenic miRNAs (oncomiRs) and tumor suppressor miRNAs [48,49].

OncomiRs stimulate tumorigenesis by downregulating tumor suppressor genes or negative regulators of growth and survival pathways. Dysregulation of oncomiRs has been implicated in enhanced cell cycle progression, inhibition of apoptosis and promotion of metastasis. Tumor suppressor miRNAs, on the other hand, prevent malignancy by targeting oncogenes or genes that promote invasion and proliferation. Their downregulation in cancer leads to uncontrolled growth and aggressive tumor behavior [47,49].

A well-studied example is the let-7 family, being among the first miRNAs identified with tumor suppressive activity. Let-7 members negatively regulate numerous oncogenes, including RAS, MYC, HMGA2, thereby inhibiting cell proliferation and stem cell potency [50]. Reduced expression of let-7 has been reported in several malignancies and associated with poor prognosis, aggressive behavior and therapeutic resistance [50,51]. This significant example shows how the balance between oncogenic and tumor-suppressive miRNAs critically shapes cancer progression and highlights their potential as biomarkers and therapeutic targets.

### 3.3. Transport and Stability

Even though miRNAs primarily carry out their functions intracellularly, they are also released into the extracellular environment and circulate in a remarkably stable form. Circulating miRNAs are protected from degradation by connection with proteins such as Argonaute 2 (Ago 2), or by encapsulation within extracellular vesicles (EVs), including exosomes and microvesicles [52,53]. These vesicles serve as carriers, enabling intercellular communication by delivering miRNAs to recipient cells where they can modulate gene expression. Additionally, miRNAs are found bound to high-density lipoproteins (HDL), thus further contributing to their stability in the bloodstream [54].

The structural protection of miRNAs makes them resistant to endogenous RNases, as well as to harsh physical conditions such as multiple freeze–thaw cycles, extremes of pH, or prolonged storage [55]. This significant resilience showcases their potential as non-invasive biomarkers accessible through liquid biopsy techniques. Beyond their diagnostic utility, EV-associated miRNAs are increasingly recognized as active factors in cancer progression, influencing the TME and processes such as angiogenesis, immune invasion, and metastasis [53,56].

### 3.4. Methods of Detection and Profiling

In order to understand the biological roles of miRNAs and their clinical applications, it is essential to ensure accurate detection and profiling. Several complementary techniques have been developed, each with specific strengths and limitations. Reverse transcription followed by quantitative polymerase chain reaction (RT-qPCR) is considered the gold standard for sensitive and specific quantification of individual miRNAs. It is particularly suitable for validating candidate biomarkers, but it has limited data throughput [57]. Microarray-based profiling enables the parallel assessment of hundreds of miRNAs in a cost-effective manner, yet it is less sensitive for low-abundance miRNAs (low concentrations within cells or biological samples) and may be affected by cross-hybridization [58]. Next-generation sequencing (NGS) provides the most comprehensive approach, allowing unbiased discovery of novel miRNAs, isoforms, and differential expression patterns with single-nucleotide resolution [59]. NGS, however, requires complex bioinformatics pipelines and remains more resource intensive. Additional methods such as digital droplet polymerase chain reaction (ddPCR) and nanotechnology-based platforms are emerging to enhance the sensitivity, precision, and reproducibility, especially in the context of liquid biopsies [60]. Overall, selecting the appropriate technique depends on the research question, sample type, and whether the goal concerns discovery, validation, or clinical application.

miRNA quantification and profiling are subject to significant variability, as not only the analytical platform (RT-qPCR, microarray, NGS, ddPCR, nanotechnology-based assays) but also the type of biological sample (plasma, serum, whole blood, exosomes, tissue lysates) and pre-analytical conditions (collection method, storage, hemolysis, platelet activation) can critically affect the results. For example, miRNA levels in plasma versus serum may differ due to the release of cellular miRNAs during clotting, and hemolysis or platelet lysis can introduce bias in measured miRNA abundances (e.g., overestimation of miRNAs abundant in red blood cells or platelets). Therefore, sample-type—dependent biases represent a major obstacle to cross-study reproducibility. It is emphasized that pre-analytical and analytical factors must be strictly controlled to ensure validity of miRNA measurements [61,62].

To mitigate these issues, standardization across all steps is vital: consistent sample collection protocols (anticoagulant choice, timing, centrifugation), quality control measures (assessment of hemolysis, RNA integrity), uniform ENA isolation methods, and normalization approaches. Only through rigorous methodological harmonization can miRNA profiling studies in uLMS produce reproducible, comparable, and clinically translatable results.

### 3.5. Clinical Utility in Oncology

The stability and accessibility of miRNAs in body fluids have placed them as promising non-invasive biomarkers in oncology. Circulating miRNAs, detectable in plasma, serum, urine, and other fluids, can expose tumor presence and biology, making them valuable for early detection, risk stratification, and disease monitoring [55]. As diagnostic tools, miRNA signatures have shown the ability to distinguish malignant from benign lesions and to differentiate between cancer subtypes with high sensitivity and specificity [63]. As for their role in prognosis, specific miRNA expression profiles correlate with survival outcomes, recurrence risk, and metastatic potential across various malignancies [64]. Additionally, miRNAs may serve as predictive biomarkers of response to treatment. Altered miRNA expression has been associated with chemotherapy resistance, targeted therapy efficacy, and immunotherapy outcomes, thereby suggesting their role in guiding individualized treatment [65]. Beyond their role as biomarkers, miRNAs as also being investigated as therapeutic agents themselves. Synthetic miRNA mimics aim to restore tumor suppressor activity, while inhibitors (antagomiRs) can silence oncogenic miRNAs. Several miRNA-based therapeutics are currently studied in clinical trials, underlining their translational potential [48].

## 4. miRNAs and uLMS

### 4.1. Dysregulated miRNAs in uLMS

Comprehensive profiling studies have identified distinct miRNA expression signatures (Table 3) that strongly differentiate uLMS from benign uterine leiomyomas, highlighting their fundamental role in tumorigenesis. A consistent finding is the significant downregulation of the miR-29 family and the miR-200 family in uLMS tissue [5,66]. The loss of function of these miRNA families is critical given their tumor suppressor abilities. miR-29b targets key oncogenic pathways like AKT3, while miR-200c inhibits epithelial–mesenchymal transition (EMT) by targeting ZEB1 [67]. Conversely, several miRNAs are frequently upregulated, thus functioning as oncomiRs. miR-21 is one of the most consistently overexpressed miRNAs in uLMS and is strongly associated with enhanced cell survival and proliferation through the inhibition of tumor suppressors like PTEN [68,69]. In a similar manner, the miR-183~96~182 cluster is upregulated, with miR182 specifically promoting metastatic behavior by targeting FOXO1 and other tumor suppressors [70,71]. These dysregulated miRNAs form interconnected networks that disrupt core oncogenic pathways, thereby contributing to the aggressive phenotype of uLMS.

### 4.2. Role in Pathogenesis and Molecular Pathways

**Figure 1:**
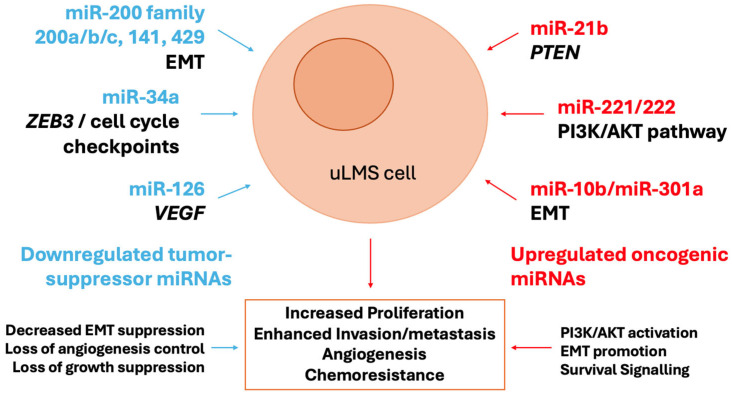
Deregulated miRNAs and their impact on uLMS biology.

The dysregulated miRNAs in ULMs are active drivers of tumorigenesis by coordinately disrupting key signaling pathways (Figure 1). A central node of convergence is the PI3K/AKT/mTOR pathway, a critical regulator of cell growth and survival. The synchronized upregulation of miR-21 (which targets PTEN, a key inhibitor of PI3K signaling) and downregulation of miR-29 (which targets AKT3) creates a powerful synergistic effect that leads to hyperactivation of this oncogenic cascade [72,73]. Furthermore, the frequent loss of the miR-200 family promotes the acquisition of a mesenchymal, invasive phenotype through the activation of the EMT transcription factor ZEB1, a direct target of miR-200c [67]. EMT is further reinforced by the upregulation of the miR-183~96~182 cluster, which enhances migratory capacity. In addition, the downregulation of the let-7 family contributes to the pathogenesis of uLMS by slowing down proliferation, as let-7 targets key oncogenes such as HMGA2 and RAS [74]. This miRNA-driven rewiring of cellular circuitry stimulates uncontrolled proliferation, evasion of apoptosis, and metastatic dissemination, hallmarks of uLMS aggressiveness.

### 4.3. Diagnostic Potential

The distinct miRNA expression profiles of uLMS hold significant promise for addressing the critical diagnostic challenges of distinguishing this malignancy from benign leiomyomas and other uterine masses. Several circulating miRNAs, including miR-1246 and miR-191-5, are significantly elevated in the serum of patients with uLMS compared with controls, suggesting their utility in as minimally invasive markers detectable through liquid biopsy approaches [75]. Such circulating markers could be particularly valuable for preoperative risk stratification when imaging and biopsy are inconclusive. On the tissue level, three-miRNA panel (miR-144-3p, miR-34a-5p, and miR-206) has been identified and has the ability to distinguish uLMS from leiomyomas with high accuracy, offering a potential molecular supplement to histopathology [76]. Additional studies also indicate that miRNA signatures may differentiate uLMS from other gynecologic malignancies, underscoring their diagnostic specificity [77]. However, most investigations to date have been limited to cohort studies and heterogenous methodologies. Therefore, larger multicenter studies are required to validate candidate miRNAs, establish standardized detection platforms, and integrate miRNA profiling into clinical routine workflow. Once confirmed, miRNA-based diagnostics may significantly reduce diagnostic uncertainty and enable earlier, more precise management of uLMS.

### 4.4. Prognostic and Predictive Value

Determining the prognosis in uLMS is extremely difficult due to its aggressive behavior and marked heterogeneity. Recent studies suggest that miRNA expression patterns may provide additional prognostic information beyond the conventional clinicopathologic parameters. For instance, the downregulation of miR-10b-5p has been shown to enhance proliferation and invasion in uLMS cell models, and its low expression correlates with more aggressive phenotypes, thus suggesting a role as a negative prognostic biomarker [78]. Tumor-suppressive miRNAs such as miR-34a and miR-181b have been reported to inhibit invasion and migration in uterine mesenchymal tumors, thereby implying that their loss may contribute to higher metastatic potential and shorter progression-free survival [79]. Furthermore, deregulation of oncogenic miRNAs, such as miR-221/222, may predict resistance to conventional chemotherapy, as demonstrated in other sarcomas and gynecologic tumors [80]. Collectively, these findings suggest that miRNA signatures may refine risk stratification and assist in identifying patients at increased risk for recurrence or poor response to therapy. Prospective validation is, however, essential before integrating these novel approaches into clinical prognostic models.

### 4.5. Therapeutic Implications

The recognition of miRNAs as key regulators in oncogenic pathways has stimulated interest in their potential as therapeutic targets in uLMS. One strategy involves the use of miRNA mimics to restore the function of tumor-suppressive miRNAs. For instance, miR-34a mimics have been shown to inhibit proliferation and invasion in preclinical sarcoma models, and similar approaches could counteract the loss of miR-34a which is frequently observed in uLMS [81]. Conversely, antagomiRs or locked nucleic acid (LNA) inhibitors can silence oncogenic miRNAs such as miR-221/222, which are overexpressed in uterine sarcomas and implicated in cell cycle dysregulation as well as chemoresistance [80]. Early preclinical studies show that blocking these miRNAs could enhance sensitivity to standard chemotherapy. An equally significant area is the development of delivery systems that ensure stability, bioavailability, and tumor targeting of miRNA-based therapies. Approaches under investigation include lipid nanoparticles, viral vectors, and engineered extracellular vesicles, which have demonstrated efficient delivery in animal models [48]. While no miRNA-based therapy has yet been clinically tested in uLMS, lessons from other solid tumors, such as phase I trials of miRNA-34a mimics in liver cancer, illustrate both the therapeutic promise of miRNAs and the challenges of toxicity and immune-related adverse effects [82]. Overall, these advances indicate that miRNA-based therapies may represent a future avenue for targeted treatment in uLMS, though significant translational barriers remain.

### 4.6. Comparison with Other Sarcomas and Gynecologic Malignancies

Comparative profiling of miRNAs across sarcomas and gynecologic malignancies has highlighted both overlapping and distinct expression patterns in uLMS. For instance, miR-221/222 overexpression, a hallmark of cell cycle dysregulation, is seen not only in uLMS but also in endometrial carcinomas and other soft tissue sarcomas, suggesting a conserved oncogenic role across mesenchymal and epithelial tumors [80]. In contrast, downregulation of miR-1 and miR-133a appears to be more specific to uLMS and may aid in distinguishing it from leiomyomas and gastrointestinal stromal tumors [83]. Larger sarcoma studies have identified recurrent deregulation of miRNAs such as miR-199a and miR-320a, yet their expression in uLMS is inconsistent, underlining the heterogeneity of this type of tumor [84]. When compared with gynecologic carcinomas, uLMS miRNA signatures often demonstrate stronger links to mesenchymal pathways such as ECM remodeling and EMT, reflecting the differences in tumor origin. These findings suggest that while some miRNAs act as pan-sarcoma drivers, others may serve as subtype-specific biomarkers, thereby providing opportunities for differential diagnosis and tailored therapy. Further integrative studies directly comparing miRNA landscapes across uterine tumors and other sarcomas will be critical to refine the diagnostic and therapeutic utility of these molecules.

## 5. Discussion and Future Perspectives

The accumulating body of evidence unequivocally demonstrates that miRNAs are integral players in the pathogenesis of uLMS. They contribute to its aggressive phenotype by coordinately dysregulating core cellular processes, including proliferation, apoptosis, and metastasis, through pathways such as PI3K/AKT, p53 signaling, and EMT. The identification of specific miRNA signatures—such as downregulation of the miR-29 and miR-200 families, together with upregulation of miR-21 and the miR-183~96~182 cluster—provides not only mechanistic insight but also tangible biomarkers with diagnostic and prognostic potential. The stability of miRNAs in circulation further leads the way to liquid biopsy approaches, which hold promise not only for preoperative diagnosis but also for longitudinal monitoring and early recurrence detection.

Despite this promising progress, the clinical translation of miRNA research in uLMS faces substantial hurdles. Technical barriers include the absence of standardized protocols for miRNA extraction, quantification, and data normalization, which impairs the reproducibility across studies. The tumor heterogeneity of uLMS further complicates the efforts to define a universal molecular signature. On the therapeutic side, even though miRNA mimics and inhibitors have shown efficacy in preclinical sarcoma models, their clinical development is hindered by challenges in targeted delivery, tissue specificity, long-term safety, and immunogenicity.

Looking forward, future research must focus on several key areas. These include validation of miRNA signatures in large, prospective, and multi-institutional cohort studies; consensus-driven methodological standardization for sarcoma miRNA analysis; and investment in advanced delivery systems, such as nanoparticles and engineered extracellular vesicles. Integration of miRNA data with genomic, transcriptomic, and proteomic networks will provide a more comprehensive map of uLMS biology, while combination therapies leveraging miRNAs as sensitizers to chemotherapy, or targeted agents may help overcome resistance to treatment. Comparative research across other sarcomas and gynecologic malignancies will clarify which miRNA alterations are uLMS-specific and which are shared, guiding both subtype-specific and pan-sarcoma strategies.

## 6. Conclusions

miRNAs have firmly established their relevance in uLMS biology and clinical management. The path forward requires coordinated, interdisciplinary, and international efforts to move from descriptive profiling toward clinical validation and therapeutic innovation. Success in these areas holds the promise of improving the dismal outcomes for patients with this rare malignancy, embedding miRNA biology into the paradigm of precision oncology for sarcomas.

## Figures and Tables

**Table 1:** Key Distinctions among Leiomyoma, STUMP ^1^, and Leiomyosarcoma.

Feature	Leiomyoma	STUMP ^1^	uLMS
** Clinical Presentation**	Abnormal uterine bleeding, pelvic pressure, infertility; usually slow growing	Similar to leiomyoma; sometimes rapid growth or atypical features	Abnormal bleeding, pelvic/abdominal pain, rapidly enlarging uterine mass, systemic symptoms in advanced disease
** Gross features**	Well-circumcised, firm, whorled cut surface	Variable; may show atypical, degenerative changes	Often large, soft, fleshy with areas of hemorrhage and necrosis
** Cytologic atypia**	Minimal to absent	Focal or moderate atypia (insufficient for uLMS diagnosis)	Diffuse, moderate to severe atypia
** Mitotic index (per 10 HPF)**	<5 (or >5 if mitotically active)	5–9 (or focal ≥ 10 with no other malignant features)	≥10, typically diffuse
** Tumor Cell Necrosis **	Absent or ischemic type only	May be absent or equivocal	Coagulative, abrupt transition viable/necrotic tissue
** Immunohistochemistry**	SMA+, Desmin+, h-caldesmon+, ER/PR usually retained	Variable ER/PR; smooth muscle markers preserved	SMA+, Desmin+, h-caldesmon+; frequent ER/PR loss
** Molecular alterations**	MED12 mutations common	Heterogenous; may harbor some atypical alterations	*TP53*, *RB1*, *ATRX* mutations frequent; high genomic instability
** Behavior**	Benign, negligible risk of recurrence (except rare variants)	Unpredictable: some recur/metastasize, most indolent	Aggressive, high recurrence and metastatic potential

^1^ STUMP: Smooth Muscle Tumors of Uncertain Malignant Potential.

**Table 2:** Reported 5-year OS ^1^, recurrence rates and common recurrence sites in uLMS by stage.

STAGE	5-Year OS ^1^	Recurrence Rate	Recurrence Sites
** I**	50–60%	50–70%	Lung, peritoneum
** II**	30–40%	60–70%	Lung, liver
** III**	<20%	70–80%	Lung, liver, peritoneum
** IV**	<15%	>80%	Multiple distant sites

^1^ OS: overall survival. Adapted data [14,33,38].

**Table 3:** Key miRNA signatures in uLMS ^1^.

miRNA	Expression in uLMS ^1^	Target Genes	Clinical Implication	References
** miR-29 family **	Downregulated	*AKT3*, *CDK6*, *ECM* ^2^ genes	Tumor suppressor, diagnostic biomarker, low expression -> poor prognosis	[72]
** miR-200 family**	Downregulated	*ZEB1*	Tumor suppressor, loss -> metastasis and poor survival	[67]
** miR-21 **	Upregulated	*PTEN*, *PDCD4*	OncomiR, diagnostic biomarker, high expression -> advanced stage	[73]
** miR-183~96~182**	Upregulated	*FOXO1*, *MITF*	OncomiR, prognostic biomarker for metastatic risk	[70]
** let-7 family**	Downregulated	*HMGA2*, *RAS*, *MYC*	Tumor suppressor, loss -> aggressive disease	[74]

^1^ uLMS: uterine leiomyosarcoma, ^2^ *ECM*: extracellular matrix.

## Data Availability

No new data were created or analyzed in this study. Data sharing is not applicable to this article.

## Abbreviations

The following abbreviations are used in this manuscript:

Ago 2	Argonaute 2
ALT	Alternative Lengthening of Telomeres
ddPCR	Digital Droplet Polymerase Chain Reaction
EMT	Epithelial-Mesenchymal Transition
ER	Estrogen Receptors
EVs	Extracellular vesicles
HDL	High-density Lipoproteins
HPF	High-power fields
LNA	Locked Nucleic Acid
miRNAs	microRNAs
MRI	Magnetic Resonance Imaging
mRNAs	Messenger RNAs
NGS	Next Generation Sequencing
oncomiRs	Oncogenic miRNAs
OS	Overall Survival
PARP	poly(ADP-ribose) polymerase
PR	Progesterone Receptors
Pre-miRNAs	Precursor miRNAs
Pri-miRNAs	Primary miRNAs
RISC	RNA-induced Silencing Complex
RT-qPCR	Reverse transcription followed by quantitative Polymerase Chain Reaction
SMA	Smooth Muscle Actin
STUMP	Smooth Muscle Tumors of Uncertain Malignant Potential
TME	Tumor Microenvironment
uLMS	Uterine leiomyosarcoma
